# DC-free Method to Evaluate Nanoscale Equivalent Oxide Thickness: Dark-Mode Scanning Capacitance Microscopy

**DOI:** 10.3390/nano14110934

**Published:** 2024-05-26

**Authors:** Mao-Nan Chang, Yi-Shan Wu, Chiao-Jung Lin, Yu-Hsun Hsueh, Chun-Jung Su, Yao-Jen Lee

**Affiliations:** 1 Department of Physics, National Chung Hsing University, Taichung 402, Taiwan; 2 Institute of Nanoscience, National Chung Hsing University, Taichung 402, Taiwan; 513excellent@gmail.com (Y.-S.W.); apple11122237@gmail.com (C.-J.L.); h44444y@gmail.com (Y.-H.H.); 3 Department of Electrophysics, National Yang Ming Chiao Tung University, Hsinchu 300, Taiwan; cjsu@nycu.edu.tw; 4 Institute of Pioneer Semiconductor Innovation, National Yang Ming Chiao Tung University, Hsinchu 300, Taiwan; yjlee1976@nycu.edu.tw

**Keywords:** scanning capacitance microscopy, equivalent oxide thickness, high-k, DC stress, mapping

## Abstract

This study developed a DC-free technique that used dark-mode scanning capacitance microscopy (DM-SCM) with a small-area contact electrode to evaluate and image equivalent oxide thicknesses (EOTs). In contrast to the conventional capacitance–voltage (C–V) method, which requires a large-area contact electrode and DC voltage sweeping to provide reliable C–V curves from which the EOT can be determined, the proposed method enabled the evaluation of the EOT to a few nanometers for thermal and high-k oxides. The signal intensity equation defining the voltage modulation efficiency in scanning capacitance microscopy (SCM) indicates that thermal oxide films on silicon can serve as calibration references for the establishment of a linear relationship between the SCM signal ratio and the EOT ratio; the EOT is then determined from this relationship. Experimental results for thermal oxide films demonstrated that the EOT obtained using the DM-SCM approach closely matched the value obtained using the typical C–V method for frequencies ranging from 90 kHz to 1 MHz. The percentage differences in EOT values between the C–V and SCM measurements were smaller than 0.5%. For high-k oxide films, DM-SCM with a DC-free operation may mitigate the effect of DC voltages on evaluations of EOTs. In addition, image operations were performed to obtain EOT images showing the EOT variation induced by DC-stress-induced charge trapping. Compared with the typical C–V method, the proposed DM-SCM approach not only provides a DC-free approach for EOT evaluation, but also offers a valuable opportunity to visualize the EOT distribution before and after the application of DC stress.

## 1. Introduction

As expected from Moore’s law, the feature size of metal–oxide–semiconductor (MOS) transistors has been reduced to 10 nm since 2016 [1]. The shrinkage of transistors enhances the functionality of integrated circuits and reduces manufacturing costs in the semiconductor industry. From a device perspective, size scaling also improves the on–off current ratio and leads to low-power and high-speed operations, thereby enhancing transistor performance [2,3,4]. The typical influential factors in size scaling are the gate length and oxide thickness [5,6,7]. Oxide–silicon and oxide–oxide interfaces strongly affect the equivalent dielectric constant of the gate oxide controlling the electrical performance of an MOS device. Therefore, the equivalent oxide thickness (EOT) is commonly used as an indicator of the electrical performance of an oxide film on silicon, especially the high-k gate oxide of metal–insulator–semiconductor devices [8,9]. Typically, the EOT for an MOS structure is extracted from capacitance–voltage (C–V) measurements obtained at various frequencies, enabling an in-depth understanding of the quality of the oxide–silicon interface [10]. If reliable C–V curves are to be obtained, the area of the contact electrode must be large and DC voltage sweeping is necessary. Current leakage and charge trapping induced by DC voltage sweeping are challenges in the acquisition of typical high-frequency C–V measurements for the evaluation of the small EOT of high-k oxide films [11,12]. Therefore, a technique to evaluate the EOT without the requirement of DC voltages must be developed.

Scanning capacitance microscopy (SCM) is a material characterization technique capable of acquiring differential capacitance signals under DC-free conditions. These signals enable the imaging of carrier distributions and p–n junctions in semiconductors such as Si, SiC, GaN, InP, and InGaAs [13,14,15,16,17]. SCM enables analog C–V measurements and provides an electrical image of a semiconductor surface. The factors influencing SCM signals have been widely studied. For instance, the photoperturbation effect induced by the laser module in an SCM system has been found to result in a false image when the system is used to map silicon-based p–n junctions [18]. This problem led to the development of dark-mode SCM (DM-SCM). The use of a high modulation voltage in SCM was discovered to lead to a distorted image during the scanning of a p–n junction [19], indicating the importance of using a low modulation voltage in SCM measurements. In a previous study, the voltage modulation efficiency for an SCM measurement was defined using a signal intensity model and the effects of the surface treatment and back-contact process used for the specimen preparation of SCM signals were studied in depth [20]. The aforementioned study found that the signal intensity model of SCM enabled a quantitative evaluation of the physical thickness of a thin insulating piece of material [20]; this result suggests a promising strategy for DC-free thickness measurements of thin insulating layers. According to [20], two thickness references can be used to precisely evaluate the physical thickness of a piece of dielectric because of the linear relationship between the SCM signal ratio and the thickness ratio [20]. This approach is promising when conducting quantitative EOT evaluations using SCM measurements. The present study employed DM-SCM to establish a DC-free method to quantitatively evaluate the EOT of a dielectric oxide film on silicon and demonstrate EOT mapping using SCM image operations.

## 2. Experimental Details

### 2.1. Specimen Preparation

**Figure 1:**
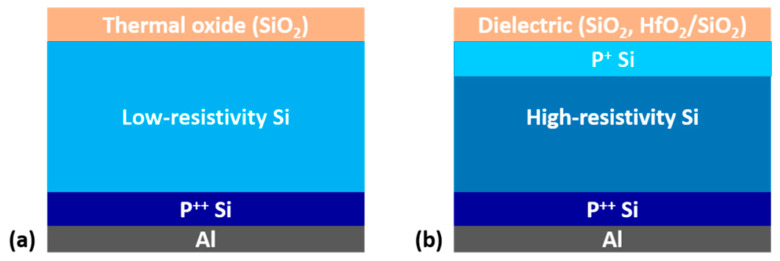
(**a**) Schematic of the layer structure for group 1 specimens. A SiO_2_ layer with a certain physical thickness was thermally grown on a low-resistivity silicon substrate with an ohmic back-contact. (**b**) Schematic of the layer structure for group 2 specimens. Two types of oxide layers—namely, a thermally grown SiO_2_ layer and a HfO_2_ film deposited on a SiO_2_ thin-film—were grown on a high-resistivity silicon substrate with a boron-implanted surface region and an ohmic back-contact.

Two groups of samples were fabricated using standard semiconductor processes and characterized using the instruments at the Taiwan Semiconductor Research Institute. The semiconductor processes adopted were cleaning, photolithography, ion implantation, thermal annealing, surface oxidation, chemical etching, and thin-film deposition. Samples with and without patterned alumina electrodes on their surface oxide were prepared for the C–V and SCM measurements, respectively. The patterned alumina electrodes had dimensions of 100 μm × 100 μm. Figure 1a shows the main structure of the group 1 samples, which were denoted D1, D2, and D3. The physical thicknesses of the thermally grown SiO_2_ films of these samples were 2.82, 4.09, and 4.85 nm, respectively. Each of these samples comprised a low-resistivity silicon substrate covered with a thermally grown SiO_2_ layer and an ohmic back-contact. The ohmic back-contact was formed via boron implantation followed by 1 s thermal annealing at 1050 °C. An alumina film was then deposited on the wafer back and subjected to rapid thermal annealing at 200 °C for 30 min. Regarding the boron implantation, the boron dose was $1\times 10^{15}$ cm^−2^ and the ion energy was 10 keV. To enable the measurement of the physical thickness of the oxide thin-films using high-resolution transmission electron microscopy (HRTEM), an electron-beam coating was produced to cover the surface oxide of each specimen with a Pt layer and a focused ion-beam system was used to prepare the cross-sections of the HRTEM specimens.

Figure 1b displays the layer structure of the group 2 samples. A high-resistivity silicon substrate was covered with two types of oxide thin-films—namely, a thermally grown SiO_2_ film and a HfO_2_ layer on an ultrathin SiO_2_ layer—and an ohmic back-contact was provided; the processes used were the same as those for the group 1 samples. The HfO_2_ layer and ultrathin SiO_2_ layer were formed via atomic layer deposition and chemical oxidation, respectively. For samples D4 and D5, the physical thicknesses of the thermally grown SiO_2_ films were 4.19 and 4.93 nm, respectively. Regarding the stacking oxide film, the physical thicknesses of the ultrathin SiO_2_ layer and HfO_2_ layer were 0.96 and 2.51 nm, respectively. After HfO_2_ deposition, 30 s annealing was performed at 400, 500, and 600 °C to obtain samples H4, H5, and H6, respectively. The area of all the SCM specimens was 4 mm^2^ to suppress stray capacitance during the SCM measurements [20]. Table 1 lists the physical thicknesses of the oxide films for the specimens and the specimen labels.

### 2.2. Instruments

**Figure 2:**
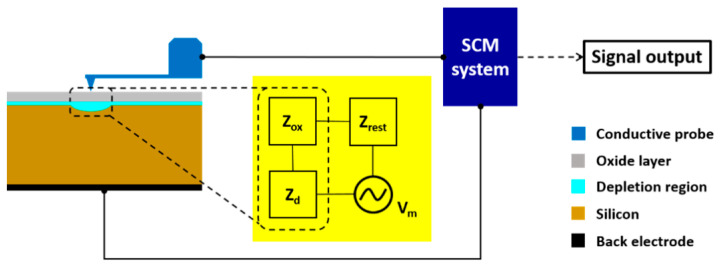
Schematic of the experimental setup for scanning capacitance microscopy (SCM) measurements. In the yellow square, three dominant impedance components are in contact with a modulation voltage source in series.

The main instruments used in this study were C–V measurement systems, an SCM system, and an HRTEM system. The C–V measurements were obtained using a 4284 LCR meter (Keysight Technologies, Inc., Santa Rosa, CA, USA) and a B1500A semiconductor device analyzer (Keysight Technologies, Inc., Santa Rosa, CA, USA) with a multifrequency capacitance measurement unit. The operation frequencies were 100 kHz, 500 kHz, and 1 MHz. In addition, a Bruker ICON scanning probe microscope with an SCM module was used to obtain the SCM measurements. The amplitude of the modulation voltage was 300 mV and the frequency was 91 kHz. A PtIr-coated Si probe tip with a force constant of 2.8 N/m and a tip radius of ~25 nm was used to acquire the SCM signals. The typical contact force used during the SCM measurements was ~35 nN. The scan rate was as slow as 0.5 Hz to minimize the tip-radius variability. To avoid photoperturbation, all SCM measurements were obtained in dark-mode. No DC bias was applied to the specimens during the SCM measurements (i.e., DC-free SCM). Figure 2 illustrates the experimental setup employed for the SCM measurements; the scheme in the yellow square in Figure 2 shows the modulation voltage source in contact with three dominant impedance components in series. To determine the physical thickness of the oxide film, a JEOL 2010F HRTEM system with an accelerating voltage of 200 kV was used to acquire the HRTEM images.

### 2.3. Method for EOT Evaluation Using DC-Free SCM

According to prior research [20], the relationship between the SCM signal intensity *S* and the modulation voltage *V*_m_ can be expressed as follows:

(1) $$S=\beta V_{m}\left(Z_{d}/Z_{t}\right),$$

where β , $Z_{d}$ , and $Z_{t}$ are the gain parameter of the SCM system, the impedance of the surface depletion region, and the total impedance, respectively. The parameter $Z_{t}$ comprises $Z_{d}$ , the oxide impedance $Z_{ox}$ , and the impedance contributed by the other series components: the conductive probe, silicon substrate, ohmic back-contact, and SCM system. The term $Z_{ox}$ represents the equivalent impedance comprising the contributions of the physical thickness of the oxide and the silicon–oxide interface. For all SCM specimens in this study, the stray capacitive impedance was negligible because the specimen area was 4 mm^2^ [20]. Of the series impedance components of $Z_{t}$ , only $Z_{d}$ is voltage-dependent. The contribution of the silicon–oxide interface is considered in $Z_{ox}$ . Equation (1) can thus be rewritten in the following form:

(2) $$S=\beta V_{m}\left(Z_{d}/(Z_{ox}+Z_{cont}\right)).$$

Samples D1–D3 were oxide films grown on identical silicon substrates with an ohmic back-contact; however, they had different uniform EOTs. The corresponding SCM signals—S1, S2, and S3, respectively—were influenced by the oxide films’ impedance and denoted as $Z_{ox1}$ , $Z_{ox2}$ , and $Z_{ox3}$ , respectively. According to Equation (2), the signal ratio S1/S2 is $\left(Z_{ox2}+Z_{cont}\right)/(Z_{ox1}+Z_{cont})$ and is denoted as $R_{s}^{12}$ . As the EOT ratio is equal to the $Z_{ox}$ ratio, the following relationship can be obtained between $R_{s}^{12}$ and the EOT ratio by defining the EOT ratio $EOT_{2}$ / $EOT_{1}$ as $R_{EOT}^{21}$ :

(3) $$R_{s}^{12}=R_{EOT}^{21}(\frac{Z_{ox1}}{Z_{ox1}+Z_{cont}})+(1-\frac{Z_{ox1}}{Z_{ox1}+Z_{cont}}).$$

**Figure 3:**
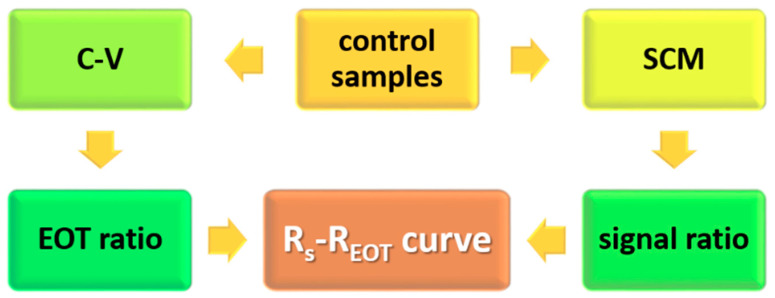
Procedure used to establish the control line for equivalent oxide thickness (EOT) evaluation.

This equation clearly indicates that the relationship between the signal ratio $R_{s}^{12}$ and the EOT ratio $R_{EOT}^{21}$ has the form of a line, the slope of which is $Z_{ox1}/(Z_{ox1}+Z_{cont})$ . The linear relationship between the signal ratio $R_{s}^{13}$ and the EOT ratio $R_{EOT}^{31}$ has the same slope. The line expressed in Equation (3) can be employed as a control line and obtained using any two reliable samples as control samples such as the three samples in group 1. The control line established using $R_{s}^{12}$ and $R_{EOT}^{21}$ can be employed to obtain EOT_3_ from the EOT ratio $R_{EOT}^{31}$ . Figure 3 illustrates the procedure used to establish the $R_{s}–R_{EOT}$ control line. During SCM measurements, DC bias must not be applied to the sample electrode.

**Figure 4:**
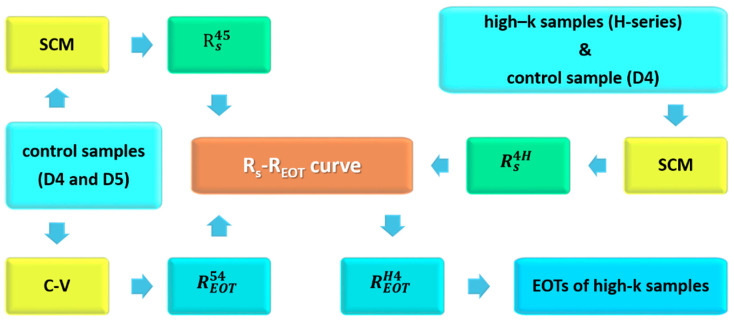
Procedure used to determine the EOT of HfO_2_/SiO_2_ films. After the *R*_S_–*R*_EOT_ linear relationship is established using two control samples, the signal ratio of a high-k sample and one control sample is employed to obtain the EOT ratio. The EOT of the HfO_2_/SiO_2_ film can then be calculated using the EOT ratio.

In principle, the EOT of a high-k oxide film on silicon (e.g., the H-series specimens) can be estimated from the EOTs of two control samples (e.g., D4 and D5). Figure 4 shows the procedure used to estimate the EOT of the H-series specimens. In this case, the $R_{s}$ – $R_{EOT}$ linear relationship for D4 and D5 could be described as follows:

(4) $$R_{s}^{45}=R_{EOT}^{54}(\frac{Z_{ox4}}{Z_{t}^{4}})+(1-\frac{Z_{ox4}}{Z_{t}^{4}}),$$

where $Z_{t}^{4}$ is the total impedance for D4 during the SCM measurements. For D4 and the H-series specimens, Equation (4) could be written as follows:

(5) $$R_{s}^{4H}=R_{EOT}^{H4}(\frac{Z_{ox4}}{Z_{t}^{4}})+(1-\frac{Z_{ox4}}{Z_{t}^{4}}).$$

The terms $R_{s}^{4H}$ and $R_{EOT}^{54}$ were obtained from the high-frequency C–V and SCM measurements, respectively, and the slope $Z_{ox4}$ / $Z_{t}^{4}$ for Equations (4) and (5) could then be obtained. Consequently, $R_{EOT}^{H4}$ could be calculated from the $R_{s}^{4H}$ value determined from the SCM measurements. Subsequently, the EOTs of the H-series specimens could be obtained by multiplying $R_{EOT}^{H4}$ by the EOT of D4.

## 3. Results

### 3.1. EOT Evaluation

#### 3.1.1. *R_s_–R_EOT_* Linear Relation

**Figure 5:**
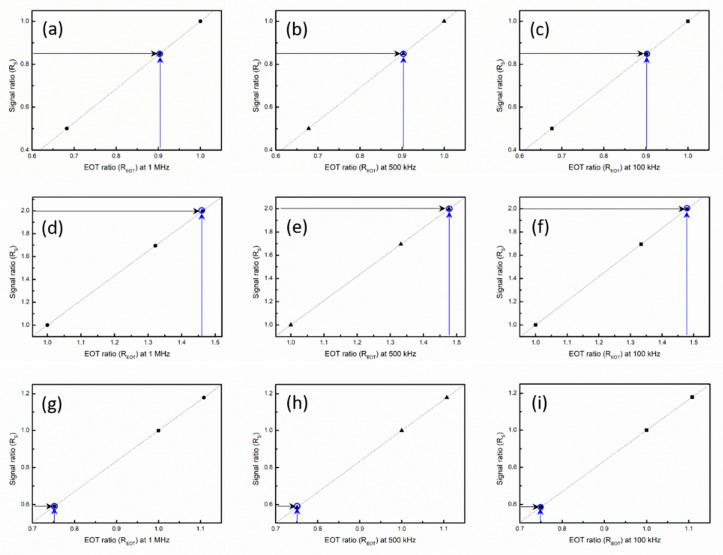
$R_{s}$ – $R_{EOT}$ control lines obtained at various frequencies. The parameters $R_{s}$ and $R_{EOT}$ were determined from SCM and capacitance (C)–voltage (V) measurements, respectively. The reference samples were D1 and D3 for the control lines in (**a**–**c**), D1 and D2 for the control lines in (**d**–**f**), and D2 and D3 for the control lines in (**g**–**i**). The black and blue arrows indicate the evaluated positions on the control lines for the SCM signal ratio and EOT ratio, respectively.

According to Equation (3), $R_{s}$ – $R_{EOT}$ linear curves corresponding with different frequencies could be obtained by using any two group 1 samples as references. The procedure illustrated in Figure 3 indicated that the C–V and SCM measurements were the first two steps required when establishing an $R_{s}$ – $R_{EOT}$ control line. For D1, D2, and D3, the average SCM signal intensities determined from a scan area of 0.7 μm × 0.7 μm were 65.584, 38.711, and 32.839 mV, respectively. The corresponding relative standard deviations (RSDs) of the SCM measurements were 2.32%, 6.27%, and 5.44%, respectively. The root mean square roughness obtained using atomic force microscopy was about 0.09 nm for all samples. For the C–V measurements conducted at 1 MHz, the EOTs of D1, D2, and D3 were 4.25, 5.62, and 6.23 nm, respectively; the corresponding RSDs of the C–V measurements were 1.38%, 2.80%, and 1.93%, respectively. For the C–V measurements conducted at 500 kHz, the EOTs of D1, D2, and D3 were 4.27, 5.69, and 6.30 nm, respectively; the corresponding RSDs of the C–V measurements were 1.43%, 2.89%, and 2.06%, respectively. For the C–V measurements conducted at 100 kHz, the EOTs of D1, D2, and D3 were 4.26, 5.68, and 6.30 nm, respectively; the corresponding RSDs of the C–V measurements were 1.43%, 2.76%, and 1.98%, respectively. Figure 5a–c show the frequency-dependent $R_{s}$ – $R_{EOT}$ control line obtained when the reference samples were D1 and D3. The black arrows indicate the position on the control line corresponding with the SCM signal ratio S1/S2 (or $R_{s}^{12}$ ). The EOT ratio $R_{EOT}^{21}$ obtained from $R_{s}^{12}$ was highly consistent with the EOT ratio $EOT_{2}$ / $EOT_{1}$ obtained from the C–V measurements, as indicated by the blue arrows. Similarly, Figure 5d–f display the $R_{s}$ – $R_{EOT}$ control line obtained when the reference samples were D1 and D2. The black arrows indicate the position on the control line corresponding with the SCM signal ratio S1/S3 (or $R_{s}^{13}$ ). The EOT ratio $R_{EOT}^{31}$ obtained from $R_{s}^{13}$ was highly consistent with the EOT ratio $EOT_{3}$ / $EOT_{1}$ obtained from the C–V measurements, as indicated by the blue arrows. Similar results are shown in Figure 5g–i. Table 2 lists the EOT values obtained from the C–V measurements and the SCM method. The percentage difference between the C–V and SCM measurements was smaller than 0.5% for all samples.

#### 3.1.2. EOT Evaluation of HfO_2_/SiO_2_/Si Structures

**Figure 6:**
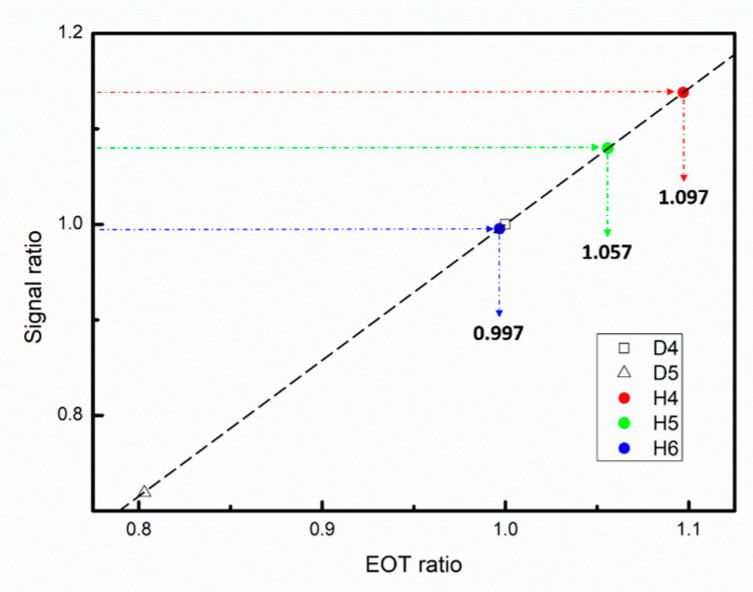
$R_{s}$ – $R_{EOT}$ control line (dashed black line) established using the reference samples D4 and D5. The blue, green, and red arrows indicate the $R_{EOT}$ values of H4, H5, and H6, respectively.

Reference samples D4 and D5 were employed to establish an $R_{s}$ – $R_{EOT}$ control line. The EOTs of high-k samples annealed at various temperatures were then evaluated using Equation (3) and the procedure illustrated in Figure 4. Figure 6 shows the $R_{s}$ values and the corresponding $R_{EOT}$ values. The EOT was calculated to be 4.03, 4.19, and 4.43 nm for H4, H5, and H6, respectively. By contrast, the EOT values extracted from the C–V measurements were 4.05, 4.22, and 4.34 nm, respectively. For H4, H5, and H6, the difference between the values obtained from the C–V and SCM measurements was 0.3%, 0.5%, and 1.2%, respectively. For the aforementioned results, the operation frequency for $R_{s}$ and $R_{EOT}$ was the same, namely, 91 kHz. In the frequency range of 100 kHz to 1 MHz, a frequency mismatch between $R_{s}$ and $R_{EOT}$ might have led to differences of up 0.88%, 2.4%, and 1.3% between the EOT values obtained from the C–V and SCM measurements for H4, H5, and H6, respectively.

### 3.2. EOT Mapping

#### 3.2.1. Thermal Oxide Subjected to Dynamic DC Stress

**Figure 7:**
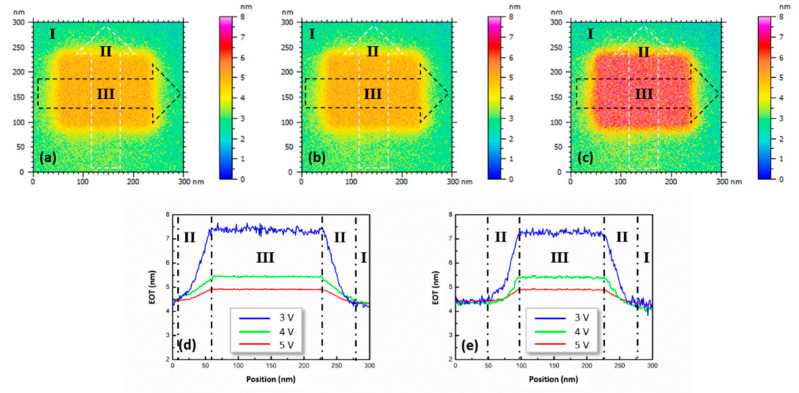
EOT images of a SiO_2_ film thermally grown on a silicon substrate when dynamic DC stress was applied at (**a**) 3, (**b**) 4, and (**c**) 5 V. The images show the nonstressed area (region I), directly DC-stressed area (region III), and region II, which was affected by a stray field around the conductive tip. The dashed black and white arrows indicate the directions of the section analyses. Results of (**d**) lateral and (**e**) vertical section analyses, which clearly indicate that the width of region II was approximately 50 nm and that the EOT distribution in region III was uniform.

To demonstrate EOT mapping, local regions of D4 were DC-stressed at various voltages (i.e., DC sample biases) using scanning with a conductive probe. Figure 7a–c present EOT images containing a region subjected to dynamic DC stress where the applied voltage was 3, 4, and 5 V, respectively. The DC-stressed area was 165 nm × 125 nm. In these EOT images, regions I and III were the areas not subjected to and subjected to DC stress, respectively. The EOT of region I was found to be approximately 4.40 nm. By contrast, the EOT of region III in Figure 7a–c was 4.87, 5.41, and 7.36 nm, respectively. Region II, which was between regions I and III, exhibited EOT variations even if the conductive tip did not directly scan this region during dynamic DC stress. In Figure 7a–c, the dashed black and white arrows indicate the directions of the section analyses. The results of the section analyses are illustrated in Figure 7d,e and indicate that the width of region II was approximately 50 nm. In addition, the EOT gradient in region II was greater when the dynamic DC stress involved higher voltage.

#### 3.2.2. High-k Oxide Subjected to Dynamic DC Stress

**Figure 8:**
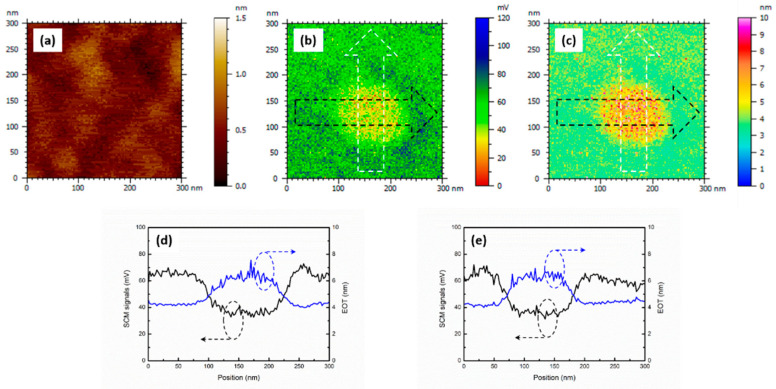
(**a**) Atomic force microscopy image, (**b**) SCM image, and (**c**) EOT image of H4 with a local area of DC stress. The DC-stressed region clearly exhibits weaker SCM signals and larger EOT values in comparison with the surrounding area. The dashed black and white arrows indicate the directions of the sectional analyses. Results of (**d**) lateral and (**e**) vertical sectional analyses, which clearly exhibit a broad peak profile, thus implying that the EOT distribution in the DC-stressed region was nonuniform.

Figure 8a–c show the atomic force microscopy image, SCM image, and EOT image of H4, respectively. The local contrast variation due to dynamic DC stress at 3 V of a sample bias on a square area of 100 nm × 100 nm (Figure 8b) was independent of the surface morphology (Figure 8a); this result implied that the dynamic DC stress did not change the oxide surface. Figure 8b,c show the SCM signals and EOT distribution for H4 before and after dynamic DC stress was applied. In Figure 8c, the area affected by the dynamic DC stress is approximately circular. The peak EOTs in the DC-stressed region and surrounding region were 7.1 and 4.1 nm, respectively. In addition, the dashed black and white arrows indicate the directions of the sectional analyses. Figure 8d,e display the results of the lateral and vertical sectional analyses, respectively, for the SCM image (Figure 8b) and corresponding EOT image (Figure 8c). These results indicated that the EOT decreased with an increase in the SCM signal intensity. The results in Figure 8d,e clearly exhibit a broad peak profile, implying that the EOT distribution in the DC-stressed region was nonuniform. The EOT image was also more straightforward than the SCM image regarding the effect of dynamic DC stress on the oxide layer.

## 4. Discussion

**Figure 9:**
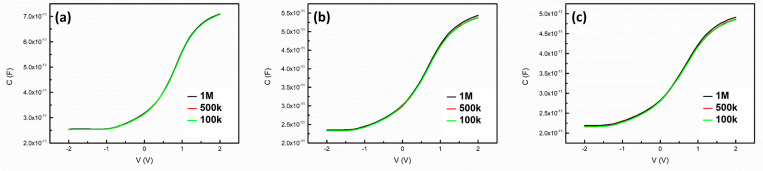
C–V curves of (**a**) D1, (**b**) D2, and (**c**) D3 over the frequency range of 100 kHz to 1 MHz. Negligible frequency dispersion can be observed in these curves.

In the proposed approach, the signal ratio and EOT ratio used to establish the control line were determined from SCM and C–V measurements, respectively (Figure 3). Thus, the signal ratio and EOT ratio could be obtained at different operation frequencies. For SiO_2_ thermally grown on Si substrates, the interface trap density has been found to be as low as 10^10^–10^11^ cm^−2^ [21]. The frequency dispersion of C–V curves in the range of 100 kHz to 1 MHz was found to be negligible in this study, as illustrated in Figure 9. Therefore, the results displayed in Figure 5 and presented in Table 2 revealed that for thermally grown SiO_2_, the SCM-based EOT evaluation produced values that were highly consistent with the results obtained from typical C–V measurements, even if a frequency mismatch occurred between $R_{s}$ and $R_{EOT}$ . In addition, the impact of tip blunting leading to tip-radius variability during scanning for the EOT evaluation was insignificant because a very small contact force, a very smooth specimen surface, a very slow scan rate, and a smaller scanning area guaranteed the reliability of the SCM measurements.

**Figure 10:**
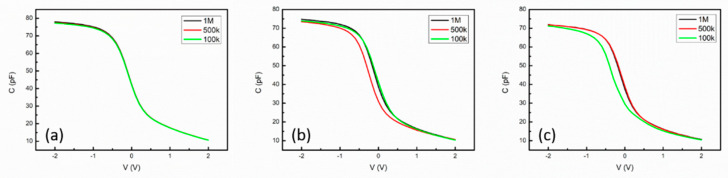
C–V curves for (**a**) H4, (**b**) H5, and (**c**) H6 in the frequency range of 100 kHz to 1 MHz. Negligible frequency dispersion can be observed in the C–V curves for H4.

A frequency mismatch between the SCM and C–V measurements should be considered in the evaluation of the EOT of the high-k oxide film because interface quality problems cannot generally be ignored. Figure 10 shows the C–V curves of H4, H5, and H6 over the frequency range of 100 kHz to 1 MHz. Negligible frequency dispersion was found in the C–V curves for H4, which implied that the HfO_2_/SiO_2_ stacked film obtained via annealing at 400 °C had an interface of extremely high quality [22]. In addition, the results displayed in Figure 6 indicate that the percentage difference in the EOT between the C–V and SCM measurements was strongly dependent on the quality of the interface in the HfO_2_/SiO_2_ stacked films, even if the same operation frequency was employed for these measurements. As the SCM measurements did not involve DC stress, the percentage differences in EOT between the C–V and SCM measurements might be attributable to the effect of DC voltage sweeping on the C–V measurements.

Finally, Figure 7 and Figure 8 indicate that the SCM method used to evaluate the EOT could be extended to image the EOT. The combination of image operations with the procedure outlined in Figure 4 resulted in a DC-free microscopic method in which an SCM image could be transformed into an EOT image by scanning using a conductive tip on the sample surface. The influence of a stray electric field surrounding the conductive tip in the EOT evaluation was directly observed in this study in the demonstration of EOT imaging (Figure 7). Upon the application of dynamic DC stress, a transition region (region II) was created between regions I and III, as shown in Figure 7. The EOT gradient in region II increased with an increase in the voltage of the dynamic DC stress because the magnitude of the stress-induced charge trapping also increased. Figure 10a clearly indicates that current leakage occurred in the inversion regime of the C–V curve, implying that the oxide quality of H4 was lower than that of D4. Thus, the charge trapping in the HfO_2_/SiO_2_ stacked film of H4 was expected to intensify upon the application of dynamic DC stress. Consequently, the EOT distribution in the DC-stressed region exhibited a broad peak profile, as shown in Figure 8d,e. For the same reason, greater charge trapping was induced by the stray electric field in the surrounding area of H4 than in the surrounding area of D4, leading to a blurred profile of the transition region for H4. Therefore, the region affected by the dynamic DC stress in Figure 8c was approximately circular. The aforementioned discussion reveals that the DC-free SCM method could be employed to image the EOT of high-k oxides on silicon and to observe EOT variations of oxide films on silicon.

## 5. Conclusions

In summary, a DC-free SCM method based on a signal intensity equation was developed in this study to precisely evaluate the EOT of oxide nanofilms on silicon, including typical thermal oxide films and a HfO_2_/SiO_2_ stacked film. The signal intensity equation represented the linear relationship between the SCM signal ratio and the EOT ratio for the EOT evaluation. When frequency dispersion was ignored, the percentage difference in the EOT values between the C–V and SCM measurements was smaller than 0.5%. In addition, EOT imaging was realized by combining the SCM method with image operations; this EOT imaging approach revealed EOT variations before and after the application of DC stresses.

## 6. Patents

An invention patent from the Republic of China (number I 832501) has resulted from the work reported in this manuscript.

## Figures and Tables

**Table 1:** Physical thicknesses of the oxide films and corresponding specimen labels.

Group 1			Group 2		
D1	D2	D3	D4	D5	H-Series
2.82 nm	4.09 nm	4.85 nm	4.19 nm	4.93 nm	2.51 nm/0.96 nm

**Table 2:** EOT values obtained from SCM and C–V measurements at various frequencies.

	**SCM**			**C–V @ 100 kHz**		
Samples	D1	D2	D3	D1	D2	D3
EOT	4.28 nm	5.68 nm	6.31 nm	4.26 nm	5.68 nm	6.30 nm
	**SCM**			**C–V @ 500 kHz**		
Samples	D1	D2	D3	D1	D2	D3
EOT	4.29 nm	5.68 nm	6.30 nm	4.27 nm	5.69 nm	6.30 nm
		**SCM**		**C–V @ 1 MHz**		
Samples	D1	D2	D3	D1	D2	D3
EOT	4.23 nm	5.63 nm	6.22 nm	4.25 nm	5.62 nm	6.23 nm

## Data Availability

The data presented in this study are available upon reasonable request from the corresponding author.
